# TE-Locate: A Tool to Locate and Group Transposable Element Occurrences Using Paired-End Next-Generation Sequencing Data 

**DOI:** 10.3390/biology1020395

**Published:** 2012-09-12

**Authors:** Alexander Platzer, Viktoria Nizhynska, Quan Long

**Affiliations:** Gregor Mendel Institute (GMI), Dr. Bohr-Gasse 3, 1030 Vienna, Austria; Email: alexander.platzer@gmi.oeaw.ac.at (A.P.); viktoria.nizhynska@gmi.oeaw.ac.at (V.N.)

**Keywords:** transposable element, NGS data, calling TEs, paired-end reads, structural variation discovery, GWAS

## Abstract

Transposable elements (TEs) are common mobile DNA elements present in nearly all genomes. Since the movement of TEs within a genome can sometimes have phenotypic consequences, an accurate report of TE actions is desirable. To this end, we developed TE-Locate, a computational tool that uses paired-end reads to identify the novel locations of known TEs. TE-Locate can utilize either a database of TE sequences, or annotated TEs within the reference sequence of interest. This makes TE-Locate useful in the search for any mobile sequence, including retrotransposed gene copies. One major concern is to act on the correct hierarchy level, thereby avoiding an incorrect calling of a single insertion as multiple events of TEs with high sequence similarity. We used the (super)family level, but TE-Locate can also use any other level, right down to the individual transposable element. As an example of analysis with TE-Locate, we used the Swedish population in the 1,001 Arabidopsis genomes project, and presented the biological insights gained from the novel TEs, inducing the association between different TE superfamilies. The program is freely available, and the URL is provided in the end of the paper.

## 1. Introduction

Transposable elements (TEs) have made themselves a great career, from being junk DNA [1] when first discovered [2], to having important roles in development [3], evolution [4,5], and disease [6] through direct genome rejoining [7], epigenetic control [8,9], or other known [10] or to-be-tested mechanisms [11].

The new quantity of next generation sequencing (NGS) data allows the discovery of structural variations (SVs) per individual and even intra-individual [12]. As TEs are an important source of SVs, their exact movements and copy number are of interest (e.g., studies [13,14,15,16]). One pitfall of TEs is their high sequence similarity, which causes alignment difficulties, especially for the short reads of most NGS platforms. This issue runs like a common thread beside the main method and analysis in this paper.

Given the difficulties of discovering TEs in general, we restricted ourselves to TEs with given sequences. Assuming the availability of a reference genome and the annotation of existing TEs in this reference genome, we developed TE-Locate, a computational tool that can call the newly-inserted copy of known TEs in sequenced individuals.

**Figure 1:**
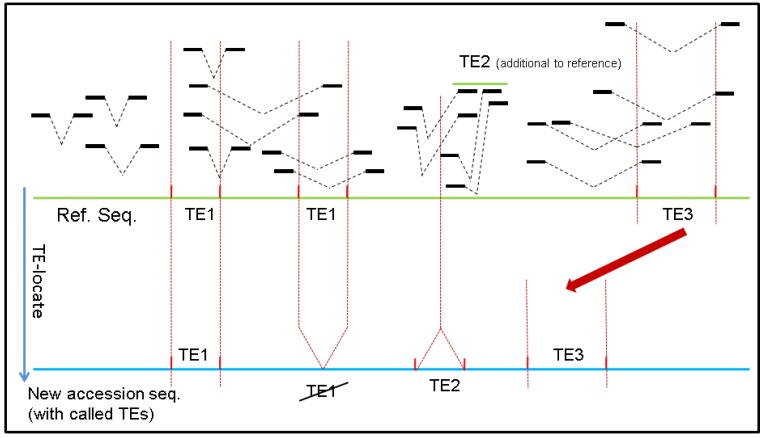
How TE-Locate makes the callings with read pairs. In this scenario one element of TE1 has vanished from one locus (while the other is retained), one TE2 was inserted, and TE3 has moved to another nearby locus (*i.e.*, cut and paste).

Two important insights into how TE-Locate functions should be noted. The first rationale underlying TE-Locate is the use of paired-end information. Although sequences of different TEs may be quite similar, the newly inserted regions should still somehow be divergent. Therefore, if a pair of reads is mapped across the breakpoint, we could observe one end of the mate-pair mapped onto the flanking sequences of the newly-inserted region with reasonably good quality, with the other end on the jumping TE (Figure 1).

**Figure 2:**
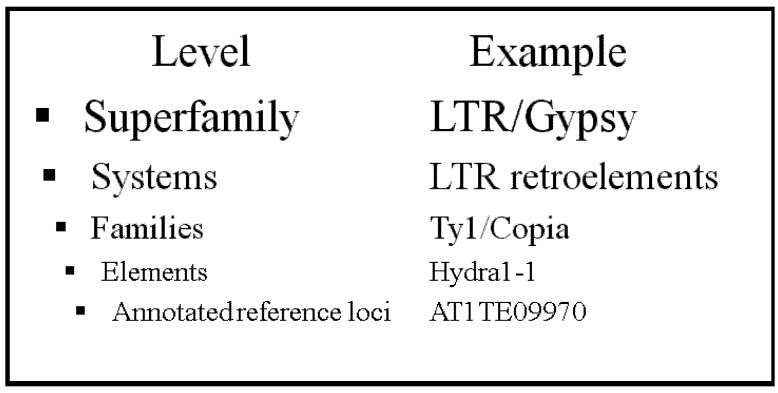
TE hierarchies in The Gypsy Database (GyDB) of Mobile Genetic Elements.

However, although we can assume the read mapped to the flanking sequence of the new regions is uniquely mapped, we may ask if the read mapped to TE itself still suffers from repetitiveness. This would result in many different mistaken TE callings in the same spot due to their similarity in sequence content. In fact, this is true, and leads to the second insight underlying TE-Locate: although different TEs from a similar template may not be easily distinguishable, one can look at the level of difference within TE families or even superfamilies (Figure 2). For example, we may be able to conclude a new TE from a particular TE family that is inserted into a certain region, without specifying what exactly the TE gene is. The level of detailed information is thereby somewhat reduced, but a more reliable result is produced. In TE-Locate, we provide different levels of abstraction so that users can balance the trade-off between specificity and reliability. 

In addition to locating new copies of TEs, TE-Locate can also be used for calling insertions of other known sequences that are not TEs. In the general case, as long as a list of known to-be-likely inserted sequences is present as a template, TE-Locate can locate their new copies in the genome of the focal individual(s). A straightforward example is positioning the insertions of a virus to the host genome [17]; a less obvious application could be to chase the known ribosomal cluster sequences in the genome [18], which is what we are attempting using *Arabidopsis* data. 

## 2. Results

### 2.1. Validation/Simulation

**Figure 3:**
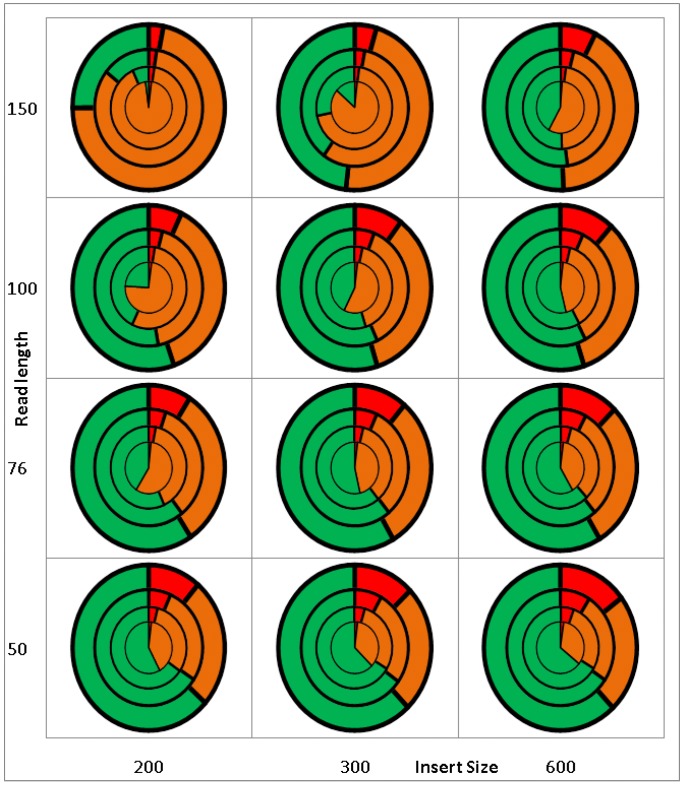
Results of TE-Locate with a virtual genome with known TEs. The X-axis denotes different insert sizes; the Y-axis denotes different read length; the concentric circles denote different coverage: from inner to outer circles, the coverages are 2×, 5×, 10× and 20× respectively. The red, orange, and green colors denote the proportion of false positives, false negatives and the rest. Here the false positive is defined as the ratio between false calls and all calls, the false negative is defined as the ratio between missing calls and all TEs inserted.

The outcome of TE-Locate is highly dependent on the aligner and the chosen hierarchy level (Figure 2). Nevertheless, we make an attempt at validation with simulated data. Firstly, a virtual reference genome is constructed starting from the *Arabidopsis thaliana* reference and its TE annotation [19]: the annotated TE regions are extracted and taken as additional sequences beside the (TE-free) chromosomes. This new reference is used later for analysis. For generation of the samples, the TE sequences are inserted back into the (TE-free) reference chromosomes, but at random locations. 500,000 SNPs (Single Nucleotide Polymorphism) (=0.4% of the whole genome) are mutated in this virtual individual genome. Based on that artificial sample, read pairs are generated with wgsim (part of Samtools [20]) for all combinations of coverages of 2×, 5×, 10× and 20×, insert sizes of 200, 300 and 600 bp (±100 bp standard deviation), and read lengths of 50, 76, 100 and 150 bp. The parameters for the real population data [21,22] which we later used for demonstrating analyses (insert size = 300 bp, read length = 76/100 bp, #SNP = 494,000, coverage = 20×) fit well to the simulations. The generated read pairs of the virtual individual genome are then aligned with BWA [23] to the virtual reference genome. The results with respect to error rates of TE-Locate with this data are shown in Figure 3. We choose superfamily as the hierarchic level. The calls are counted as correct if the right superfamily is called within 3-fold of the standard deviation of the read pair’s insert size. The results are divided into chromosomal arms and pericentromeric regions (there are nearly no calls in the centromeres). Only the arms regions are depicted in Figure 3; the other diagram for pericentromeric regions, which shows slightly higher error rates, is the Supplementary Figure S1. One can see several trends in Figure 3: the False Positives (FP) decrease and the False Negatives (FN) increase with higher read lengths. This is expected, since very small TEs are missing when the read length decreases, at least with our chosen aligner. An efficient aligner that is able to deal with split reads would be helpful. There is an opposite effect with larger insert sizes and higher coverage (if the thresholds of calling the variants are fixed for any coverage). We also tried the same simulated data with BreakDancer [24], and depicted results in the Supplementary Figures S2 and S3. TE-Locate clearly outperforms BreakDancer at calling TEs. However, we do acknowledge that TE-Locate leverages TE annotations and uses hierarchy levels that general SV tools such as BreakDancer do not.

### 2.2. Real Data

To demonstrate the tool and some subsequent analysis, we applied it to NGS data of ~200 Swedish *Arabidopsis thaliana* lines sequenced in our group [25], which is part of the 1,001 genomes project [21,22]. The terms ‘population’, ‘individuals’, and ‘real data’ later in the text refer to this source.

**Figure 4:**
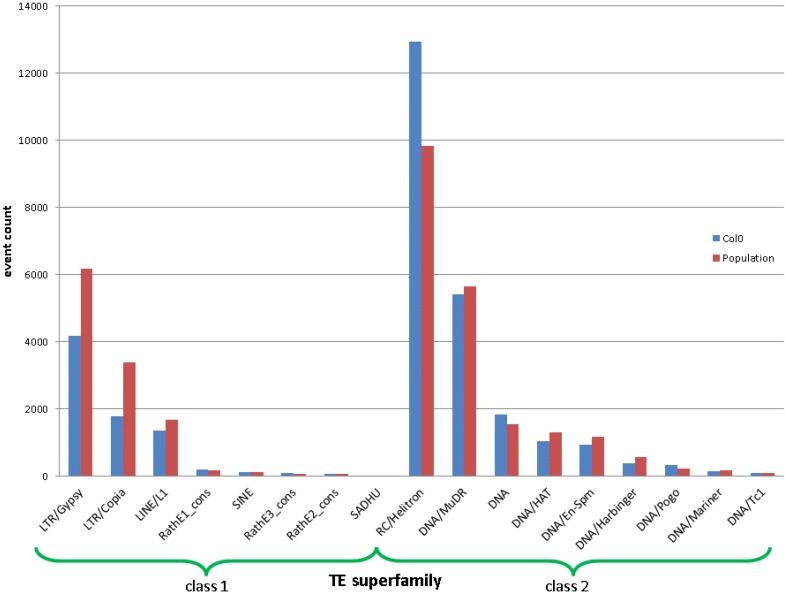
The event counts per TE superfamily annotated in the reference (blue) and newly discovered from the population. An event for the population is counted if it occurs in any individual. Class I and II TEs (“copy-paste” and “cut-paste”) are depicted separately.

In total, we called about 40,000 TEs in the population on the superfamily level (on other hierarchical levels, it called other quantities of events). By contrasting the number of TE events called and that are annotated in the reference, we see a clear difference between Class I and II (“copy-paste” and “cut‑paste”) TEs (see Figure 4). 

**Figure 5:**
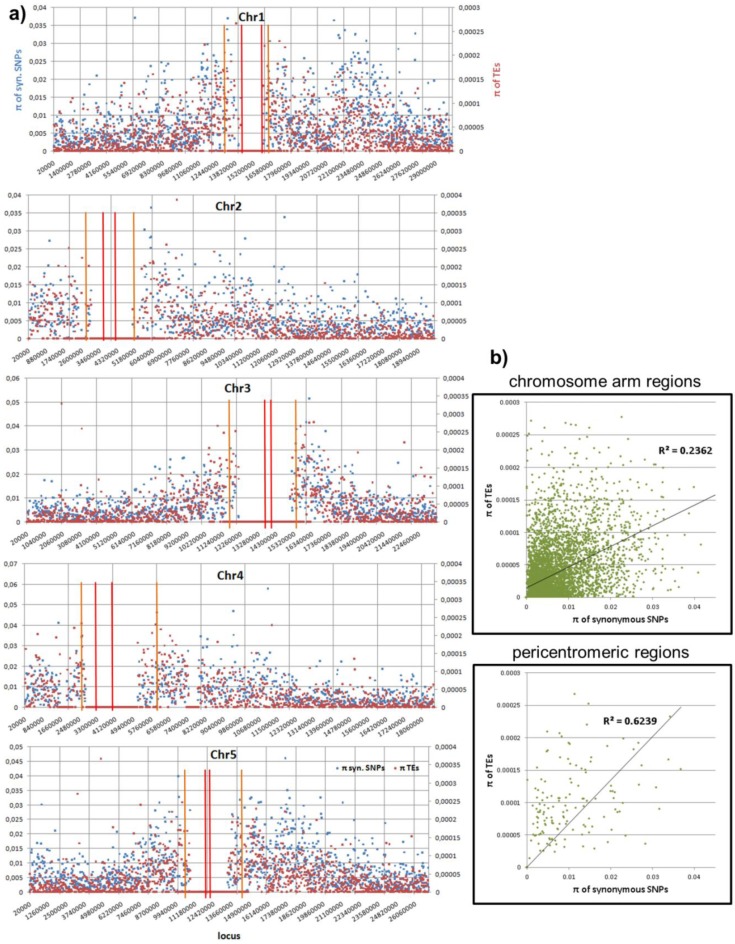
Distribution of polymorphism in terms of pair-wise difference p (in terms of the number of events without being weighted by the lengths) of the TE calls in the population against p of SNPs. Both p are computed with a window size of 20 Kb and normalized to 1 bp. (**a**) The p distribution in the chromosomes. We use red and orange bars to indicate the centeromeric and pericentromeric regions. (**b**) The correlation between TE and SNP p’s in both chromosomal arms and pericentromeric regions. If there is not even a single event in one of both windows (TE or SNP), this locus is skipped. Both correlations are highly significant (*p*-value = 0 due to machine precision).

For comparative purposes, the distribution of polymorphism in terms of pair-wise difference, p, is shown in Figure 5 for TEs and for SNPs. We found that the polymorphism of SNPs is correlated to the density of new TEs (Figure 5b) in both chromosomal arms and pericentromeric regions, which might indicate an interesting mutation or selection mechanism, if not simply an effect of a deeper coalescence time.

**Figure 6:**
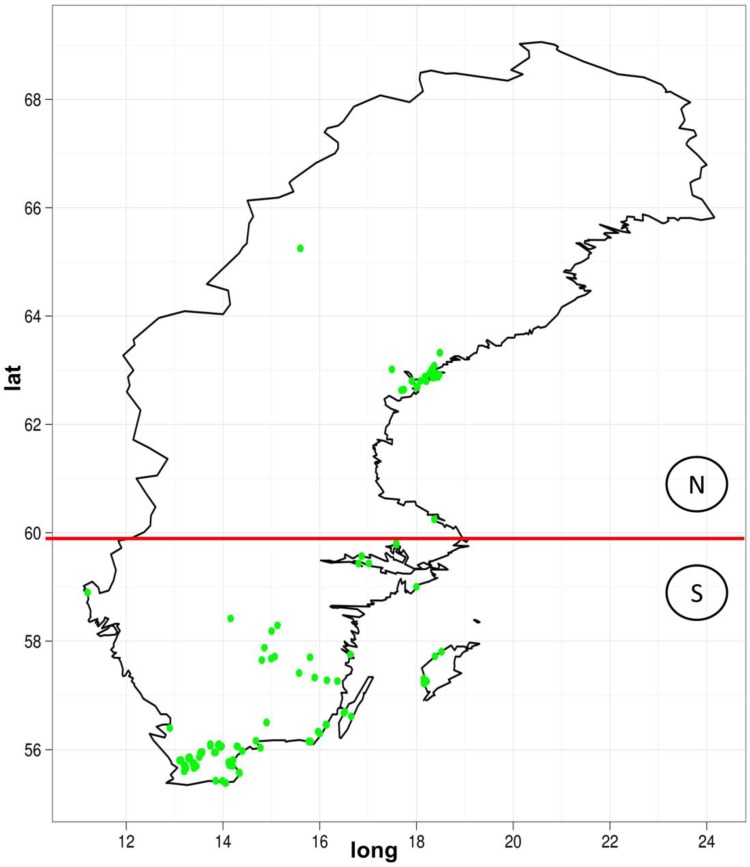
The geographic distribution of the *Arabidopsis thaliana* lines used for our analysis. The red line indicates the border between the later-used north and the south class.

**Figure 7:**
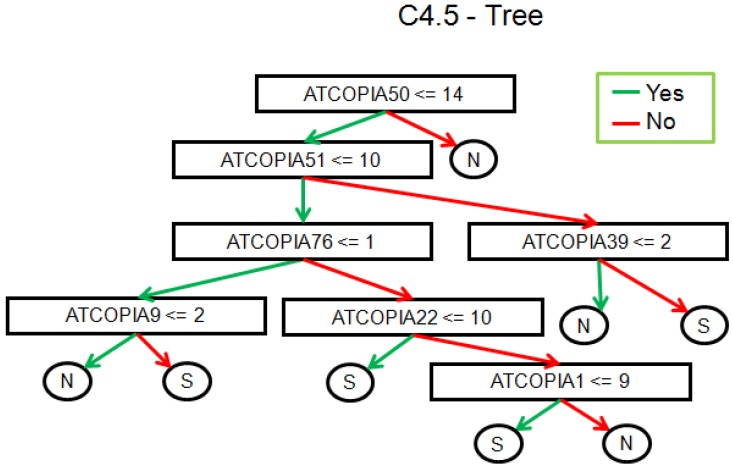
Result of the C4.5 algorithm for a classification of north *versus* south individuals with respect to their TE-family copy number. 92.5% of the individuals are correctly classified at the 10× cross-validation.

We also looked for the distribution of the copy numbers to the geographic location. The sequenced samples were divided up between the north and south of Sweden (Figure 6). The question here is whether this classification could be replicated by observing the TE variations. Based on TE-Locate results, we tried several machine learning techniques (with Weka [26]). On the superfamily level there was no result better than chance at 10× cross-fold validation. On the TE-family level, there are good classifications with a true prediction rate of 92%–98% and a lower limit *i.e*., zero ratio of 71% (zero ratio = the ratio of the more frequent class). The result of the C4.5 algorithm [27] is shown in Figure 7. With respect to the true prediction rate, this is not the best model, but trees are easier to interpret than, for example, the weights of SVMs (Support Vector Machine) [28]. As one can see in this tree, although all TE‑families were used as variables, only *Copia* families are enough to sufficiently split the classes. We did not go into detail on why the copy numbers of *Copia* families are clearly different between north and south; the simplest explanation could be merely a temperature dependency in them (see the related, but not so recent [29,30]).

**Figure 8:**
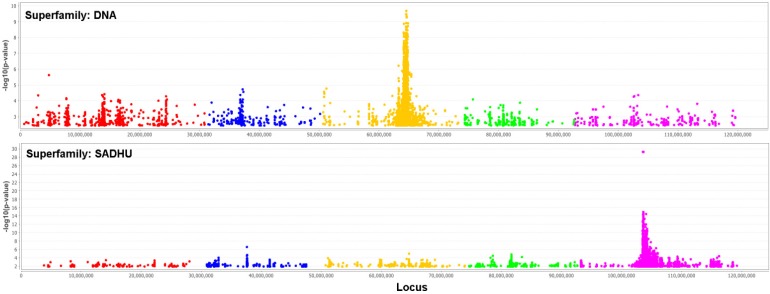
Manhattan plot of logged *p*-values of association between the SNPs and the TE copy number. The chromosomes are sequential in different colors. The upper plot uses the DNA TE-superfamily as phenotype, the lower the TE-superfamily SADHU. The Bonferroni threshold is 2.5 × 10^−7^.

We performed genome-wide association studies (GWAS) using the 4 million SNPs from the sequences as genotype, and each of the 18 TE superfamilies copy number as phenotype. The question for this analysis is, how much of the variation in TE copy numbers could be explained by the genotype. We used a mixed model [31] to control population structure and Bonferroni correction to control an inflated significance level due to multiple-test issues. Two of these GWAS with many significant SNPs are shown in Figure 8. As expected, there are many significant SNPs located in TEs themselves and unfortunately nearly none in (well-annotated) genes. An exception is one significant SNP in the auxin response factor-12 gene (AT1G34310) for the copy number of RathE3. 

**Figure 9:**
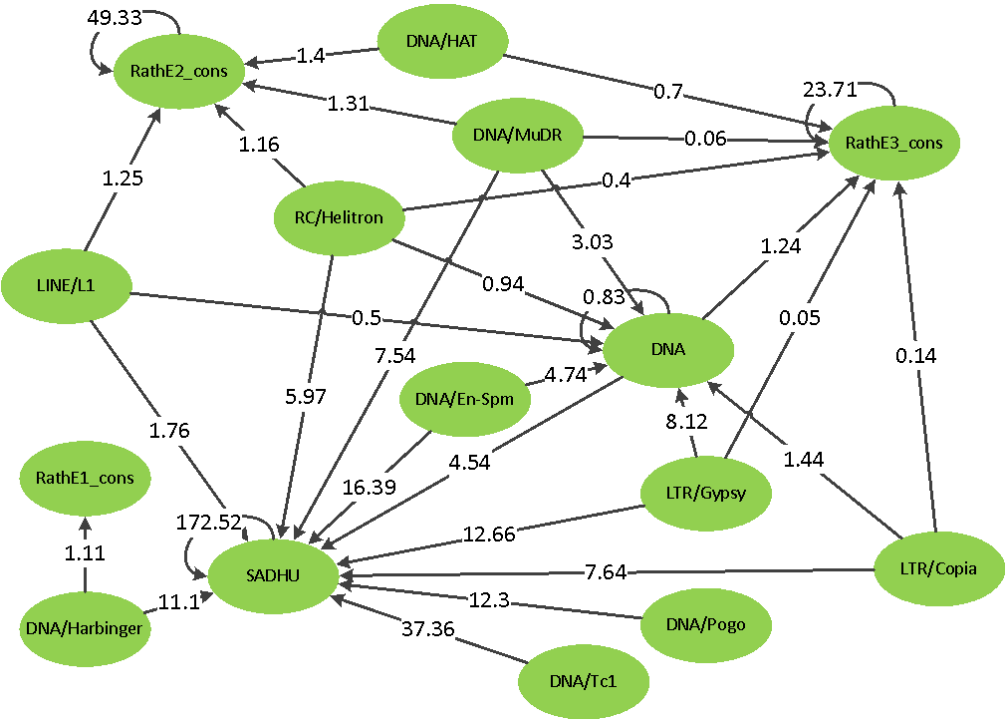
The SNP to copy number hierarchy from GWAS. The arrows indicate that the SNPs located in the superfamily on the blunt side of the arrow are significantly associated with the copy number of the superfamily on the side of the arrowhead. The number within the arrow is the number of SNPs normalized by the total length of TEs in the corresponding superfamily. There were no cases of arrows traveling in both directions.

It is remarkable that most of the significant SNPs for a superfamily are located in another superfamily. It is not clear whether this could be a problem of a too-high similarity between the superfamilies or a non-optimal separation. However, if one of these issues is causing the effect, we should have observed a symmetrical relationship between the pair of superfamilies: if SNPs associated with superfamily A are located in superfamily B, then we should also observe SNPs associated with superfamily B located in superfamily A. However, what we observed is an asymmetric hierarchy (Figure 9): it is never the case that if one superfamily has significant SNPs in another, that this is also present in the reverse case. It would be interesting to investigate the biology of this observation.

## 3. Methods

TE-Locate assumes that the user has paired-end reads. Before running TE-Locate, the read pairs are aligned with any aligner producing a BAM/SAM file (e.g., BWA [23], Smalt [32], or Segemehl [33]). With the previously prepared annotation, TE-Locate calls the TE as shown in Figure 1. TE-Locate will identify and collect all mate-pairs that have one end mapped inside a TE and the other end mapped with good quality to any region outside all TEs. By clustering all the evidential reads, the new copy of TE will then be reported. To leverage the population sharing that is crucial for structural variant callings [34], the tool is written to act on all individuals in the population at once. In this manner, individuals with very low coverage at a particular region can take advantage of other individuals when there is a genuine event also called by other good coverage individuals. 

The results are reported in two files: one is a CSV file in which the have-or-have-not information for all individuals and all events is provided. In a separate information file, TE-Locate also provides a summary of more detailed event information (features of the TE, the number of supporting reads, *etc.*) An example output is shown in Table 1; the columns are explained in detail in Table 2.

**Table 1:** Example output of TE-Locate.

chr	loc	len	event_type_ref	non_ref_counts	anc_status	read_pair_support	<unused>...	call_method	Orientation	#pPairs	#iPairs	new/old
1	5421	7679	TE+DNA/MuDR/DNA/MuDR	5	N	15	|	PairEndTE	inverse	4	11	new
1	16726	3890	TE+RC/Helitron/RC/Helitron	171	N	900	|	PairEndTE	uncertain			old
1	20843	1292	TE+RC/Helitron/RC/Helitron	3	N	63	|	PairEndTE	inverse	20	43	new
1	11897	79	TE+LTR/Copia/LTR/Copia	55	N	69	|	PairEndTE	uncertain			old
1	22277	1736	TE+DNA/MuDR/DNA/MuDR	7	N	15	|	PairEndTE	inverse	6	9	new
1	42355	10046	TE+RC/Helitron/RC/Helitron	4	N	11	|	PairEndTE	parallel	10	1	new
1	42210	4671	TE+DNA/MuDR/DNA/MuDR	5	N	11	|	PairEndTE	inverse	1	10	new
1	50968	651	TE+LTR/Gypsy/LTR/Gypsy	6	N	10	|	PairEndTE	parallel	9	1	new
1	52425	382	TE+LTR/Copia/LTR/Copia	2	N	26	|	PairEndTE	inverse	1	25	new
1	70064	4814	TE+LTR/Copia/LTR/Copia	1	N	19	|	PairEndTE	inverse	0	19	new
1	71152	799	TE+LTR/Copia/LTR/Copia	1	N	31	|	PairEndTE	parallel	31	0	new
1	55676	900	TE+DNA/HAT/DNA/HAT	174	N	2133	|	PairEndTE	uncertain			old
1	77569	831	TE+RC/Helitron/RC/Helitron	178	N	1661	|	PairEndTE	uncertain			old
1	76844	656	TE+LINE/L1/LINE/L1	75	N	753	|	PairEndTE	uncertain			old
1	84679	12225	TE+LTR/Gypsy/LTR/Gypsy	7	N	12	|	PairEndTE	parallel	10	2	new
1	91443	7263	TE+LTR/Gypsy/LTR/Gypsy	6	N	13	|	PairEndTE	parallel	11	2	new
1	116237	2941	TE+LTR/Copia/LTR/Copia	1	N	57	|	PairEndTE	parallel	47	10	new
1	129878	5185	TE+LTR/Copia/LTR/Copia	4	N	23	|	PairEndTE	parallel	23	0	new
1	154331	87	TE+LINE/L1/LINE/L1	89	N	138	|	PairEndTE	uncertain			old
1	192934	593	TE+RC/Helitron/RC/Helitron	177	N	1915	|	PairEndTE	uncertain			old

**Table 2:** Description of the TE-Locate output.

Column	Description
**chr**	Locus
**loc**	
**len**	The length of the corresponding reference event.
**event_type_ref**	The class of this event annotated (resp. the item/TE)
**non_ref_counts**	The number of individuals sharing this event.
**anc_status**	Unused
**read_pair_support**	The total number of all supporting read pairs of all individuals.
**bp_range1**	Unused...
**bp_range2**	
**four_gamete_left**	
**four_gamete_right**	
**call_method**	For TE-Locate, here is written ‘PairEndTE’, used if merged with other data in this format.
**Orientation**	‘parallel’, ‘inverse’ or ‘uncertain’: The orientation according to the reference sequence.
**#pPairs**	The number of read pairs supporting parallel orientation. Not used if the orientation is ‘uncertain’.
**#iPairs**	The number of read pairs supporting inverse orientation. Not used if the orientation is ‘uncertain’.
**new/old**	‘new’ or ‘old’. ‘old’ if the item is called at the locus in the reference; ‘new’ otherwise. Note that at higher hierarchical levels, all locations of this item are meant, e.g., any Copia called at a Copia locus in the reference is called ‘old’ as the item’s name is the only distinction.

In the real data analysis presented in this paper, the reference sequence and the TE annotations are taken from TAIR [19] in .fasta and .gff formats respectively. The *Arabidopsis thaliana* lines are sequenced by Illumina GAII as well as by HiSeq 2000 with paired-end reads 2 × 76 bp or 2 × 100 bp. The coverage ranges from 10× to 70×. More details of the dataset will be published soon and can be downloaded from the 1,001 genomes project public website [22].

The hierarchical levels of TE families are from the Gypsy Database—GyDB [35] (Figure 2). The hierarchical level should be high enough to ensure that no very similar sequences are present at different items, but low enough to have a good resolution. Most of the demonstration analysis uses the superfamily and family level. 

## 4. Discussion and Conclusions

TE-Locate is a flexible tool to call known sequences of a reference in new individuals. This is particularly interesting for TEs. The theoretical computational complexity is O(n*log(n)), where *n* is the number of reads. In practice, we observed that the implementation is sufficiently efficient, at least for our deeply-sequenced *Arabidopsis* lines. In our real data, TE-Locate needed much less computational time than the initial preprocessing of the data (mapping reads, *etc.*). Although the implementation is not parallelized, no GPGPU (General-purpose computing on graphics processing units) is used and the code is written in Perl and Java.

The current initial release of TE-Locate runs fast and its algorithm is rather straightforward. Many extensions are possible. One immediate extension is to include indel callings from various sources, perhaps also combined with graphs from *de-novo* assembly. We could also count negative support (=contradicting read pairs) and evaluate the optimal set in contradictory cases. Finally, it may be beneficial to combine with split read alignments [36] and/or develop an efficient aligner for this [37].

Not all the possible extensions will necessarily have a positive effect, at least if the thresholds for trade-offs are not chosen carefully. An example would be the trade-off between negative and positive support and the weight of split-reads against read pairs. The computational complexity will likely increase, especially if it is to find an optimal set or combination.

TE-Locate is a nice complement to other tools [38] for a similar purpose. T-lex [39] uses single split reads and only checks whether the reference loci are present or not; REPET [40], RECON [41], and TESeeker [42] call new TE sequences without leveraging existing annotations; TE-HMM [43] analyzes genomes itself to discover TEs without using read-level information. Also, all above‑mentioned tools do not take advantage of paired-end information, which is not ideal for most ongoing NGS projects in which the paired-end reads will be generated. Various indel calling tools [44] are also beneficial to TE analysis, since TEs can also be considered merely as ordinary indels. The program is freely available online [45].

## Supplementary Files

Supplementary File 1 DOCX-Document (DOCX, 720 KB)
