# Supplementary material for: TE-Locate: A Tool to Locate and Group Transposable Element Occurrences Using Paired-End Next-Generation Sequencing Data
Source: Biology (Basel). 2012 Sep 12;1(2):395–410. doi: 10.3390/biology1020395 (PMC4009769; doi:10.3390/biology1020395)

**Supplimenary Figures**

**Supplimenary Figure S1.** The error rate of TE-Locate in pericentromeric regions. The X‑axis denotes different insert sizes; the Y-axis denotes different read length; the concentric circles denote different coverage: from inner to outer circles, the coverages are 2×, 5×, 10× and 20× respectively. The red, orange, and green colors denote the proportion of false positives, false negatives and the rest. Here the false positive is defined as the ratio between false calls and all calls, the false negative is defined as the ratio between missing calls and all TEs inserted.


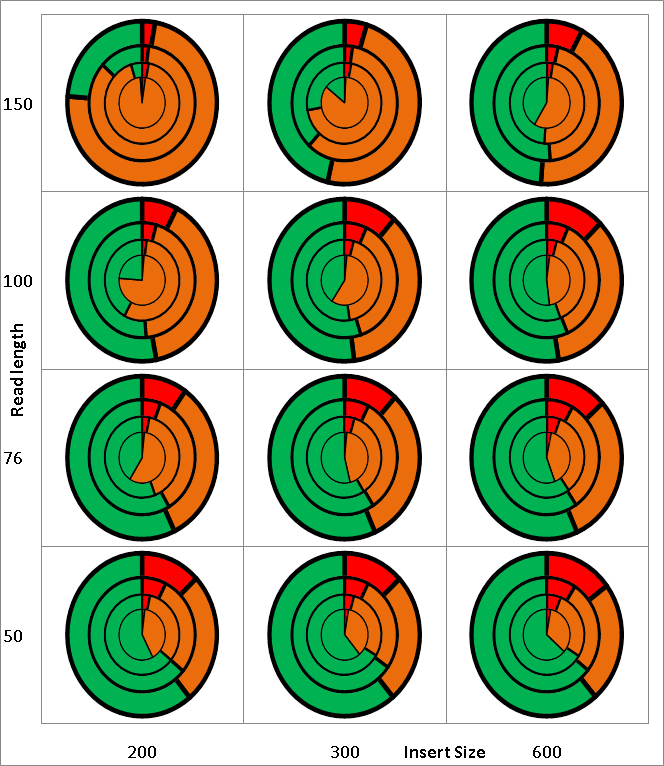


**Supplimenary Figure S2.** The error rate of BreakDancer in chromosomal arm regions. The X-axis denotes different insert sizes; the Y-axis denotes different read length; the concentric circles denote different coverage: from inner to outer circles, the coverages are 2×, 5×, 10× and 20× respectively. The red, orange, and green colors denote the proportion of false positives, false negatives and the rest. Here the false positive is defined as the ratio between false calls and all calls, the false negative is defined as the ratio between missing calls and all TEs inserted.


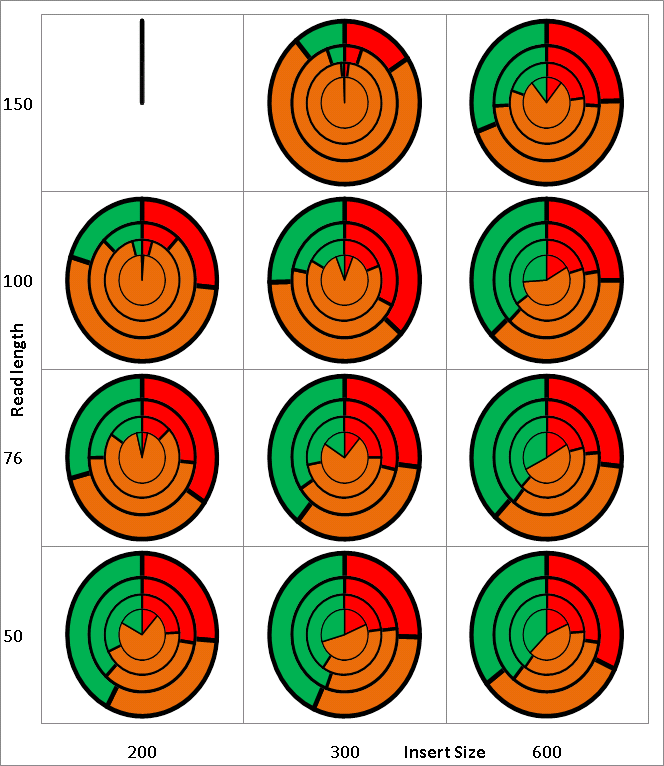


**Supplimenary Figure S3.** The error rate of BreakDancer in pericentromeric regions. The X-axis denotes different insert sizes; the Y-axis denotes different read length; the concentric circles denote different coverage: from inner to outer circles, the coverages are 2×, 5×, 10× and 20× respectively. The red, orange, and green colors denote the proportion of false positives, false negatives and the rest. Here the false positive is defined as the ratio between false calls and all calls, the false negative is defined as the ratio between missing calls and all TEs inserted.


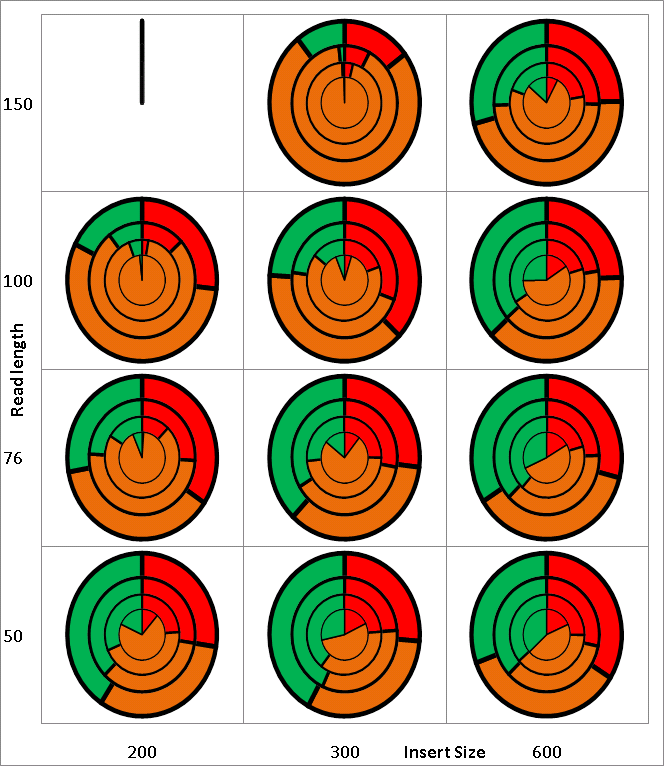

Supplement: Supplementary File 1 — DOCX-Document (DOCX, 720 KB) [file biology-01-00395-s001.docx]
